# Effect of proton pump inhibitors on occlusion of lumen-apposing metal stents and rate of endoscopic necrosectomies: a Europe-wide multicenter cohort study

**DOI:** 10.1055/a-2569-7056

**Published:** 2025-05-08

**Authors:** Jacob Hamm, Alzbeta Busana, Ahmad Amanzada, Alexander Arlt, Thomas Asendorf, Samantha Carswell, Ulrike Denzer, Louis Elsing, Fabian Frost, Lucia Guilabert, Karim Hamesch, Marcus Hollenbach, Péter Hegyi, Alexander Kleger, Jan Krivinka, Lumir Kunovsky, Christian Meinhardt, Veit Phillip, Sophie Schlosser-Hupf, Simon Sirtl, Lukas Welsch, Julian Cardinal von Widdern, Albrecht Neesse, Christoph Ammer-Herrmenau, PROTOCOL working group, Georg Beyer, Alicia Dürr, Premysl Falt, Christoph Gerst, Albrecht Hoffmeister, Péter Hegyi, Thomas Kohlmann, Richard Knoop, Belen Martinez-Moreno, Julia Mayerle, Patrick Michl, Tobias Mollnow, Martina Müller, Sanjay Pandanaboyana, Golo Petzold, Jonas Rosendahl, Lucas Alexander Schulte, Hans Seifert, Emanuel Steiner-Gager, Sabrina Sulzer, Ondrej Urban, Vincent Dansou Zoundjiekpon, Volker Ellenrieder

**Affiliations:** 1 Department of Gastroenterology, Gastrointestinal Oncology and Endocrinology, University Medical Center Goettingen, Goettingen, Germany; 2 Department of Gastroenterology, University Hospital Oldenburg, Oldenburg, Germany; 3 Department of Gastroenterology, Israelitisches Krankenhaus, Hamburg, Germany; 4 Department of Medical Statistics, University Medical Center Goettingen, Goettingen, Germany; 5 HPB and Transplant Unit, Freeman Hospital, Newcastle Upon Tyne, United Kingdom; 6 Department of Gastroenterology, University Hospital Marburg, Marburg, Germany; 7 Department of Oncology, Gastroenterology, Hepatology and Pneumology, University Hospital Leipzig, Leipzig, Germany; 8 Department of Medicine A, University Medicine Greifswald, Greifswald, Germany; 9 Department of Gastroenterology, Hospital General Universitario Dr. Balmis, ISABIAL (Instituto de Investigación Sanitaria y Biomédica de Alicante), Alicante, Spain; 10 Medical Clinic III, Gastroenterology, Metabolic Diseases and Intensive Care, University Hospital RWTH Aachen, Aachen, Germany; 11 Department of Gastroenterology, University Hospital Heidelberg, Heidelberg, Germany; 12 Translational Pancreatology Research Group, Interdisciplinary Center of Excellence for Research Development and Innovation, University of Szeged, Szeged, Hungary; 13 Institute for Translational Medicine, Medical School, University of Pécs, Pécs, Hungary; 14 Institute of Pancreatic Diseases and Centre for Translational Medicine, Semmelweis University, Budapest, Hungary; 15 Institute of Molecular Oncology and Stem Cell Biology, University Hospital Ulm, Ulm, Germany; 16 Division of Interdisciplinary Pancreatology, Department of Internal Medicine I, Ulm University Hospital, Ulm, Germany; 17 2nd Department of Internal Medicine – Gastroenterology and Geriatrics, University Hospital Olomouc, Faculty of Medicine and Dentistry, Palacky University Olomouc, Olomouc, Czech Republic; 18 Department of Surgery, University Hospital Brno, Faculty of Medicine, Masaryk University, Brno, Czech Republic; 19 Department of Gastroenterology and Digestive Endoscopy, Masaryk Memorial Cancer Institute, Brno, Czech Republic; 20 Department of Clinical Medicine II, Technical University of Munich, TUM School of Medicine and Health, TUM University Hospital, Munich, Germany; 21 Department of Internal Medicine I, Gastroenterology, Hepatology, Endocrinology, Rheumatology and Infectious diseases, University Hospital Regensburg, Regensburg, Bavaria, Germany; 22 Department of Medicine II, University Hospital LMU Munich, Munich, Germany; 23 Department of Gastroenterology, Diabetology and Infectiology, Klinikum Hanau, Hanau, Germany; 24 Department of Internal Medicine I, Martin Luther University Halle-Wittenberg, Halle, Germany; 25 Department of Scientific Law, University Medical Center Goettingen, Goettingen, Germany; 26 Institute for Community Medicine, University Hospital Greifswald, Greifswald, Germany; 27 Population Health Sciences Institute, Newcastle University, Newcastle upon Tyne, United Kingdom; 28 Department of Gastroenterology, District Hospitals, Günzburg-Krumbach, Germany; 29 Department of Internal Medicine II, Gastroenterology and Hepatology, Karl Landsteiner University of Health Sciences, University Hospital St. Pölten, St. Pölten, Austria

## Abstract

**Background** Lumen-apposing metal stents (LAMS) are widely used to drain walled-off necrosis (WON). LAMS occlusion is a significant clinical problem and identification of risk factors for LAMS occlusion could contribute to novel preventive strategies. A previous study suggested contradictory effects of proton pump inhibitors (PPIs) on occlusion and necrosectomy rates.

**Methods** We conducted a Europe-wide multicenter retrospective cohort study assessing WONs drained by LAMS. The primary aims were to assess the strength of association between PPI intake and LAMS occlusion and necrosectomy rates, respectively. The secondary aim was to assess the strength of association between PPI intake and other LAMS-associated complications. Multiple mixed-effects models were used to control for possible confounding covariates.

**Results** 893 patients with 967 LAMS from 17 centers were included. After excluding incomplete datasets and patients who took PPIs intermittently, 768 LAMS remained. The overall occlusion rate was 28.0 %. Most occlusions occurred within 10 days. Most patients received PPIs continuously (n = 577 vs. no intake n = 191). In patients who did not use PPIs continuously, lower rates of LAMS occlusion (odds ratio [OR] 0.61, *P* = 0.04) and necrosectomies (incidence rate ratio 0.8, *P* = 0.006) were observed. A post hoc analysis exhibited a dose- and compound-dependent effect of PPI intake on necrosectomy rate. No increase in other complications in the non-PPI group, such as bleeding events (OR 1.14) were observed.

**Conclusion** PPI intake was associated with higher rates of LAMS occlusion and necrosectomy.

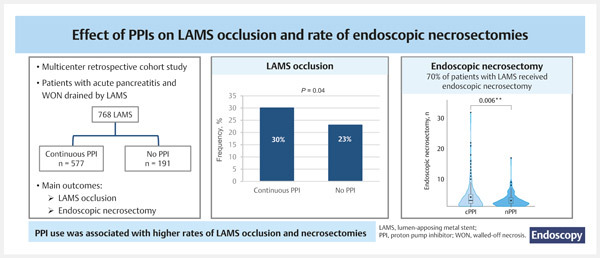

## Introduction

The development of walled-off necrosis (WON) occurs in 5 %–10 % of all patients with acute pancreatitis 1 . Significantly increased mortality has been reported for patients with infected necrosis (28 %) compared with sterile necrosis (13 %), which underlines the need for effective drainage strategies to resolve complicated WON 2 .

Since their introduction in 2012, lumen-apposing metal stents (LAMS) have been widely used for the drainage of WON in patients with acute pancreatitis 3 4 . Overall rates of adverse events after LAMS placement have been reported to range from 12.4 % to 38.6 % 5 6 .

LAMS occlusion is a relevant clinical problem that may prevent resolution of WON and can lead to superinfection and subsequent septic episodes 7 . Previous meta-analyses have reported a wide range of occlusion rates, from 3.8 % to 24.3 % 8 9 . To date, only a few factors that potentially influence the LAMS occlusion rate have been discussed, such as the pre-emptive placement of double-pigtail stents through the LAMS. However, the evidence for the benefit of placing double-pigtail stents to prevent occlusion is conflicting and thus their value is debated repeatedly 6 9 10 . Moreover, the prevalence of LAMS occlusion-related complications, such as progression or superinfection of WON, has not yet been well investigated.

Particularly important for effective resolution of WON is endoscopic necrosectomy performed through the LAMS. Although endoscopic necrosectomy is not required in all WON drained with LAMS, on average 3–5 endoscopic necrosectomies are performed via LAMS 11 12 . However, endoscopic necrosectomy bears a considerable rate of procedural adverse events, with a bleeding risk of 5.2 % per procedure per patient 11 . Moreover, patients undergoing endoscopic necrosectomy are at a significantly higher risk for overall adverse events compared with patients who are treated conservatively or only receive drainage without endoscopic necrosectomy 13 .

A previous study reported conflicting results concerning the impact of proton pump inhibitors (PPIs) on adverse events after LAMS placement. It was suggested that concomitant intake of PPIs could on the one hand entail a lower LAMS occlusion rate and on the other hand a higher rate of endoscopic necrosectomy for WON resolution 12 .

Therefore, we conducted a large real-world Europe-wide multicenter retrospective cohort study in which the primary aims were to assess the strength of association between PPI intake and the rates of LAMS occlusion and endoscopic necrosectomies, respectively. The secondary aim was to assess the strength of association between PPI intake and other LAMS-associated complications.

## Methods

### Study design

Patients aged ≥ 18 years who received LAMS for drainage of WON between 2012 and March 2024 were eligible for enrollment. Patients with placement of LAMS into pseudocysts or postoperative pancreatic fistulae instead of WONs were excluded. All cases were entered into REDCap anonymously and chronologically from the first time the LAMS was placed into a WON at the respective center to address selection bias. If a patient received more than one LAMS, all information was stored under the same record identification number. The REDCap database was provided and maintained by the Department of Medical Statistics of the University Medical Center Goettingen. Datasets with missing information on PPI intake, LAMS occlusion, or endoscopic necrosectomy were excluded from the analysis.

### Definitions

For the different modes of PPI intake, continuous PPI intake was defined as at least one dose of PPI per patient per day. For PPI dosage, 20 mg/d omeprazole, 20 mg/d esomeprazole, 40 mg/d pantoprazole, 30 mg/d lansoprazole, and 20 mg/d rabeprazole were defined as standard dosages. For a standardized assessment of complications after LAMS placement, partial LAMS occlusion was defined as any occlusion of less than 100 % of the LAMS lumen. Total occlusion was defined as an occlusion of the entire lumen. Gastrointestinal (GI) bleeding was defined as an event that led to either a blood transfusion or an intervention to stop the bleeding, such as embolization.

### Outcomes

The primary aims of this retrospective cohort study were to assess the strength of association between PPI intake and the rates of LAMS occlusion and endoscopic necrosectomies, respectively. As outcomes, the rate of LAMS occlusion and the rate of endoscopic necrosectomy, as well as the time to occlusion, were assessed in patients receiving continuous PPI therapy (cPPI group) and in those not receiving PPI therapy (nPPI group). In a first post hoc analysis, the strength of associations between PPI intake and partial/total LAMS occlusion rates was assessed. In a secondary post hoc analysis, the strength of associations between the type of PPI, dose of PPI, timepoint of PPI intake, and PPI intake regimen (continuous vs. intermittent), and LAMS occlusion as well as the endoscopic necrosectomy rate was tested. The secondary aim was to test the strength of association between PPI intake and other LAMS-related complications. As outcomes, GI bleeding rates (defined as a clinically significant bleeding requiring either blood transfusion or intervention), WON progress detected on cross-sectional imaging, superinfection of the WON, sepsis events, and length of hospital stay were assessed in the cPPI and nPPI groups.

Ethical approval was provided by the institutional review board of each participating center (initial approval from University Medical Center Goettingen, protocol number 1/2/23). It was not possible to involve patients or the public in the design, conduct, or reporting, or dissemination plans of our study.

### Statistical analysis

Based on a power calculation performed on a smaller in-house dataset from the University Medical Center Goettingen investigating PPI intake and LAMS occlusion rates (see **Supplementary methods** in the online-only Supplementary material), we aimed to include a total of 640 patients (560 in the cPPI group and 80 in the nPPI group). Eventually, 967 LAMS from 893 patients were included by 17 European centers from 6 European countries.

Data analysis was performed using R (version 4.2.3 or later; R Foundation for Statistical Computing, Vienna, Austria). Multiple mixed-effects models were applied, with specifications based on the data structure. Record identification number and years of LAMS placement were treated as random effects. For binary dependent variables (e. g. LAMS occlusion), a multiple logistic mixed-effects model was used. For nonbinary discrete variables, a cumulative link mixed-effects model was applied. Count data were modeled according to observed overdispersion (Poisson or negative binomial) and analyzed using multiple negative binomial mixed-effects models. In addition to the variable of interest (PPI regimen), potential confounding covariates were defined a priori and included as fixed effects in the models: age, sex, body mass index, type of pancreatitis (acute pancreatitis, recurrent acute pancreatitis, chronic pancreatitis), type of LAMS, LAMS diameter, route of LAMS placement, pre-emptive coaxial pigtail stent placement through the LAMS, and the intake of other gastric pH modulators. The competing risks cumulative incidence was calculated using the tidycmprsk package (R Foundation for Statistical Computing). *P* value was assessed by Gray’s test 14 . Results were considered statistically significant if the two-sided *P* value was < 0.05. For pairwise comparisons, *P* values were adjusted using the Tukey method. Adjusted *P* values are reported. If the number of events was too small to perform a reliable mixed-effects model, a logistic regression with Firth’s correction was applied to avoid overfitting, and only unconditional odds ratios (ORs) and 95 %CIs were reported.

## Results

### Association of PPI intake and LAMS occlusion rate

**Fig. 1:**
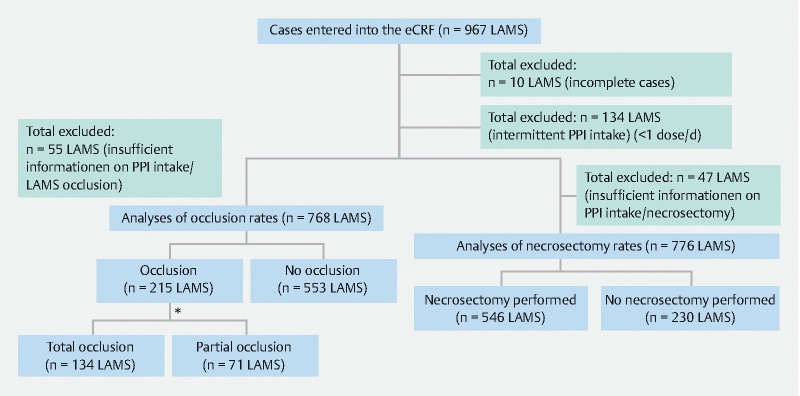
Strengthening the reporting of observational studies in epidemiology (STROBE) flow chart for primary aims. eCRF, electronic case record form; LAMS, lumen-apposing metal stent; PPI, proton pump inhibitor. *Information on the extent of occlusion was missing for 10 LAMS.

In total, 893 patients with 967 LAMS were included in the REDCap database. A total of 10 cases were excluded because of incomplete datasets. A further 55 datasets were excluded because of unavailable data on PPI intake and/or LAMS occlusion, as well as 47 datasets because of unavailable data on PPI intake and/or endoscopic necrosectomy. Additionally, all datasets in which PPIs were used intermittently (less than one dose per day) were omitted from the analysis (n = 134) ( Fig. 1 ).

**Fig. 2:**
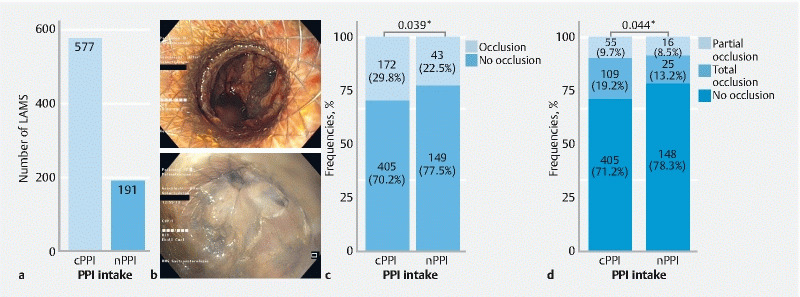
Continuous intake of proton pump inhibitors (PPI) was associated with a higher rate of lumen-apposing metal stent (LAMS) occlusion. **a** Number of LAMS in patients who were prescribed PPIs continuously (cPPI) or not at all (nPPI). **b** Example images of LAMS with no occlusion (upper panel) and total occlusion of the LAMS lumen (lower panel). **c** Overall occlusion rates for cPPI and nPPI groups. **d** Occlusion rates stratified by partial and total occlusions for cPPI and nPPI intake, respectively. * *P* < 0.05 using multivariable logistic mixed-effects model and cumulative link mixed models.

Of all LAMS (n = 768) in patients with sufficient information on PPI intake, the majority were placed in patients receiving continuous PPI therapy (cPPI, n = 577, 75.1 %) and 191 (24.9 %) were in patients who did not receive PPIs (nPPI) ( Fig. 2 **a** ). In 215 (28.0 %) of all LAMS placed, an occlusion occurred. Most occlusions were detected during gastroscopy (97.2 %) and only a minority using other modalities ( **Table 1 s** ). To associate the PPI intake regimen with LAMS occlusion, we fitted a multiple logistic mixed-effects model including factors potentially affecting the LAMS occlusion rate. As random effects we included the record identification and the years of LAMS placement as a surrogate for experience with LAMS. Strikingly, our data revealed a significantly higher overall LAMS occlusion rate in the cPPI group compared with the nPPI group (OR 0.61, *P =* 0.04) ( Table 1, Fig. 2 **b,c** ). Most LAMS occlusions occurred within 10 days after placement. Cumulative incidence plot and Gray’s test confirmed significant differences between PPI intake regimens from the above-mentioned model ( *P =* 0.048) ( **Fig. 1 s** ). Intriguingly, the occlusion occurred significantly later in the nPPI group.

**Table 1:** Association of proton pump inhibitor intake with rate of lumen-apposing metal stent occlusion controlled for potential confounders using a multiple logistic mixed-effects model.

Variables	Event rate, n/N (%)	OR	95 %CI	*P* value 1
PPI intake				
Continuous intake	166/565 (29.4)			
No intake	41/188 (21.8)	0.61	0.38–0.98	0.04*
Sex				
Female	67/235 (28.5)			
Male	140/518 (27.0)	1.04	0.70–1.54	0.85
Type of pancreatitis				
Acute pancreatitis	197/627 (31.4)			
Recurrent acute pancreatitis	4/42 (9.5)	0.28	0.09–0.84	0.02*
Chronic pancreatitis	6/84 (7.1)	0.17	0.07–0.42	< 0.001***
Type of LAMS 2				
Hot Axios	140/511 (27.4)			
Axios	26/82 (31.7)	1.22	0.6–2.23	0.51
Spaxus	6/32 (18.8)	0.89	0.33–2.46	0.83
Hot Spaxus	11/35 (31.4)	1.10	0.47–2.57	0.82
Nagi	1/6 (16.7)	0.25	0.02–3.47	0.30
Hanaro	8/52 (15.4)	0.71	0.30–1.66	0.43
Other	11/27 (40.7)	2.25	0.75–6.80	0.15
Not available	4/8 (50.0)	0.20		
Pigtail stent through LAMS				
No	177/563 (31.4)			
Yes	30/190 (15.8)	0.42	0.25–0.71	< 0.001***
Access route				
Transgastric	181/699 (25.9)			
Transduodenal	8/31 (25.8)	1.07	0.43–2.68	0.88
Not available	18 /21 (85.7)			
LAMS diameter, mm				
15–20	153 /561 (27.3)			
> 10– < 15	21/102 (20.6)	1.43	0.77–2.65	0.31
Not available	33/89 (37.1)			
Age, years				
18–29	9/28 (32.1)			
30–49	45/191 (23.6)	0.53	0.21–1.36	0.19
50–70	120/394 (30.5)	0.76	0.31–1.86	0.54
> 70	33/140 (23.6)	0.49	0.18–1.29	0.15
BMI, kg/m ^2^				
< 18	2/18 (11.1)			
18–25	67/279 (24.0)	1.31	0.25–6.75	0.75
26–35	111/395 (28.1)	1.41	0.27–7.27	0.68
> 35	27/61 (44.3)	2.99	0.54–16.7	0.21
Other gastric pH modulators				
Yes	8/25 (32.0)			
No	199/728 (27.3)	0.95	0.30–2.94	0.93

BMI, body mass index; LAMS, lumen-apposing metal stent; OR, odds ratio; PPI, proton pump inhibitor.

1 Wald’s test was performed to test significances (* *P* < 0.05, *** *P* < 0.001). The following parameters were used as random effects: patient identification and years of LAMS placement.

2 Hot Axios, Axios (Boston Scientific, Marlborough, Massachusetts, USA); Spaxus, Hot Spaxus, Nagi (Taewoong Medical Co., Goyang, South Korea); Hanaro (M.I. Tech, Pyeongtaek, South Korea).

By applying a cumulative link mixed-effects model we performed a post hoc analysis differentiating between occurrence of total and partial occlusion rates. We observed a significantly higher rate of LAMS occlusion in the cPPI group (total occlusion 19.2 %, partial occlusion 9.7 %) compared with the nPPI group (13.2 % vs. 8.5 %, respectively; OR 0.63, *P* = 0.04) ( Fig. 2 **d** , **Table 2 s** ).

To further delineate the effects of concomitant PPI intake on LAMS occlusion, we assessed which type and dose of PPI were administered in a second post hoc analysis. Moreover, we investigated whether PPIs were prescribed prior to or after LAMS placement, were stopped actively, were taken intermittently (less than one dose per day), or whether the PPI dose was changed during LAMS placement. To account for these factors, we performed a multiple logistic mixed-effects model in a second post hoc analysis of our dataset. In the multiple analysis, lansoprazole was associated with a significantly higher occlusion rate (OR 14.2, *P* = 0.002), and intermittent PPI intake was associated with a significantly lower occlusion rate (OR 0.42, *P* = 0.005) ( **Table 3 s** ).

### Association of PPI intake and endoscopic necrosectomy rate

**Fig. 3:**
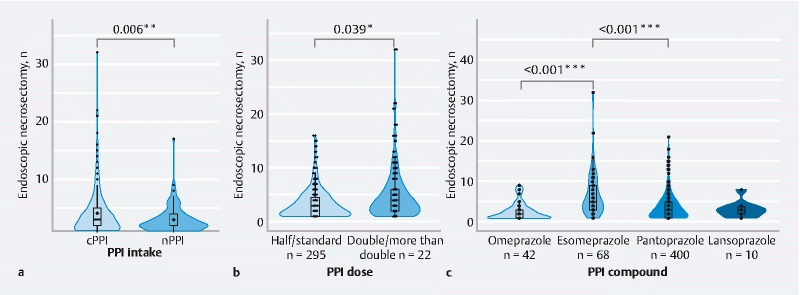
Continuous intake of proton pump inhibitors (PPIs) was associated with an increased rate of endoscopic necrosectomies in a dose- and compound-dependent manner. Violin-/boxplots displaying numbers of endoscopic necrosectomies depending on: **a** PPI intake; **b** PPI dose; and **c** PPI compound. * *P* < 0.05, ** *P* < 0.01, *** *P* < 0.001 using a multiple negative binomial mixed-effects model. cPPI, continuous PPI intake; nPPI, no PPI intake.

After excluding unavailable data on PPI intake and endoscopic necrosectomies, 546 (70.4 %) applied LAMS were identified with at least one endoscopic necrosectomy. In order to investigate the influence of PPI intake on the endoscopic necrosectomy rate while controlling for potential confounding variables, we performed a multiple negative binomial mixed-effects model, again taking into account the factors that were used for the above-mentioned model for occlusion. Remarkably, and in line with our findings regarding the occlusion rate, we observed a significantly higher number of endoscopic necrosectomies in the cPPI group compared with the nPPI group (IRR 0.8, *P* = 0.006) ( Table 2, Fig. 3 **a** ). To investigate the association with PPI intake regimen in more detail, we used the multiple negative binomial mixed-effects model to assess the effect of different PPI-related factors. We observed statistically significant changes in the endoscopic necrosectomy rates between different dosages (half/standard vs. double/more than double) (IRR 1.17, *P* = 0.039) ( Fig. 3 **b** , **Table 4 s** ) and between different PPI compounds. For the latter, we performed a pairwise comparison with *P* value adjustment. Interestingly, we discovered that intake of esomeprazole was associated with a significantly higher endoscopic necrosectomy rate compared with pantoprazole (IRR 1.56, *P* < 0.001) and omeprazole (IRR 2.06, *P* < 0.001) ( Fig. 3 **c** , **Table 5 s** ). None of the patients received rabeprazole.

**Table 2:** Association of proton pump inhibitor intake with endoscopic necrosectomy rates controlled for potential confounders using a multiple negative binomial mixed-effects model.

Variables	IRR	95 %CI	*P* value 1
PPI intake			
Continuous intake	–	–	
No intake	0.80	0.68–0.94	0.006**
Sex			
Female	–	–	
Male	1.07	0.93–1.23	0.35
Type of pancreatitis			
Acute pancreatitis	–	–	
Recurrent acute pancreatitis	0.65	0.41–1.02	0.06
Chronic pancreatitis	0.48	0.33–0.68	< 0.001***
Type of LAMS 2			
Hot Axios	–	–	
Axios	0.85	0.66–1.10	0.21
Spaxus	0.89	0.66–1.22	0.48
Hot Spaxus	0.77	0.54–1.08	0.12
Nagi	0.52	0.22–1.22	0.13
Hanaro	0.77	0.58–1.02	0.07
Other	1.28	0.84–1.93	0.25
Not available	1.11		
Pigtail stent through LAMS			
No	–	–	
Yes	1.37	1.17–1.61	< 0.001***
Access route			
Transgastric	–	–	
Transduodenal	0.71	0.49–1.02	0.06
Other access	0.78	0.25–2.44	0.67
Not available	1.02	0.70–1.47	0.93
LAMS diameter, mm			
15–20	–	–	
> 10– < 15	0.64	0.49–0.83	< 0.001***
Not available	0.94		
Age, years			
18–29	–	–	
30–49	1.02	0.73–1.43	0.92
50–70	1.05	0.76–1.44	0.77
> 70	0.88	0.62–1.25	0.48
BMI, kg/m ^2^			
< 18	–	–	
18–25	1.36	0.68–2.72	0.38
26–35	1.06	0.53–2.11	0.87
> 35	1.39	0.68–2.82	0.36

BMI, body mass index; LAMS, lumen-apposing metal stent; IRR, incidence rate ratio; PPI, proton pump inhibitor.

1 Wald’s test was performed to test significances (** *P* < 0.01, *** *P* < 0.001). The following parameters were used as random effects: patient identification and years of LAMS placement.

2 Hot Axios, Axios (Boston Scientific, Marlborough, Massachusetts, USA); Spaxus, Hot Spaxus, Nagi (Taewoong Medical Co., Goyang, South Korea); Hanaro (M.I. Tech, Pyeongtaek, South Korea).

### Association of PPI intake and other LAMS-related adverse events

As the secondary aim of this study, we assessed the occurrence of other LAMS-related complications in both the cPPI and nPPI groups. GI bleeding rates, WON progress detected on cross-sectional imaging, superinfection of the WON, sepsis events, and length of hospital stay were assessed as outcomes. The number of events in the nPPI group was too small to perform a multiple logistic mixed-effects models. A logistic regression with Firth’s correction was used and thus only an OR with 95 %CI is provided for this sub-analysis. We observed a comparable rate of GI bleeding events in both groups (OR 1.14, 95 %CI 0.61–2.02). For progression of the WON detected on cross-sectional imaging, we observed a lower rate in the nPPI group (OR 0.35, 95 %CI 0.04–1.47). Moreover, we observed comparable rates of superinfected WONs and sepsis events in the cPPI compared with the nPPI group (OR 0.75, 95 %CI 0.29–1.7 and OR 1.29, 95 %CI 0.51–2.97, respectively) ( **Table 6 s** ). Additionally, a multiple negative binomial mixed-effects model revealed no significant differences regarding duration of indwelling LAMS (IRR 0.91, *P* = 0.20) ( **Table 7 s** ). Most LAMS were removed within 35 weeks after placement ( **Fig. 2 s** ).

## Discussion

The potential effect of PPIs on LAMS occlusion has been debated among experts; however sufficient data are lacking. Mechanistically, it is believed that the higher gastric pH after intake of PPIs could lead to a higher rate of LAMS occlusion by less gastric acid flowing into the LAMS and the necrotic cavity leading to less chemical corrosion of the necrotic debris. However, thus far, only one smaller retrospective cohort study published by Powers et al. in 2019 investigated the role of PPIs in patients with LAMS 12 . Intake of PPIs was associated with a significantly lower LAMS occlusion rate, whereas the number of endoscopic necrosectomy sessions was significantly higher in patients who received PPIs. However, important factors such as the dosage, type of PPI, continuous/intermittent intake, or intake of other gastric pH modulators were not assessed in the study. Moreover, potential confounding factors were not taken into account.

By accounting for confounders in multiple mixed-effects models, our data showed that the LAMS occlusion and endoscopic necrosectomy rates were significantly higher in the cPPI group compared with the nPPI group. Moreover, we observed that occlusion events occurred earlier in the cPPI group, as revealed by cumulative incidence of occlusion. The observed increase in occlusions and endoscopic necrosectomies in the cPPI group was compound and dose dependent. For other LAMS-related complications, we observed a similar risk of significant GI bleeding events between the groups and a higher rate of WON progression on imaging in the cPPI group. 

Compared with the estimated prevalence of PPI use in outpatients (7 %–9 %), we observed a drastically higher PPI use in patients receiving LAMS (75.1 %) 15 16 . The overall LAMS occlusion rate observed within our study (27.9 %) was similar to that of previous results from meta-analyses showing occlusion rates of up to 24.3 % 9 12 . The high number of patients in our cohort who received PPIs, as well as the considerably high occlusion rate in patients receiving LAMS, underline the clinical importance of our data.

One hypothesis for the effects observed could be that a reduction in gastric acid following PPI intake could lead to higher occlusion rates. When comparing the efficiency of gastric pH suppression by different PPI compounds, it is generally accepted that all PPIs are comparably potent when normalized to omeprazole equivalents necessary to elevate the gastric pH above 4 15 . However, at the standard dosages for the different PPI compounds as defined in our study (20 mg/d omeprazole, 20 mg/d esomeprazole, 40 mg/d pantoprazole, 30 mg/d lansoprazole, and 20 mg/d rabeprazole), lansoprazole, esomeprazole, and rabeprazole had omeprazole equivalents of 27 mg, 32 mg, and 36 mg, respectively, whereas pantoprazole had an omeprazole equivalent of 9 mg 15 17 . Thus, at these dosages, lansoprazole, esomeprazole, and rabeprazole are more effective at pH suppression than omeprazole and pantoprazole. Indeed, we observed the highest occlusion rate in patients receiving lansoprazole and significantly more endoscopic necrosectomies in the esomeprazole group compared with the pantoprazole and omeprazole groups. Further supporting this hypothesis, we also observed a dose- and compound-dependent effect for the endoscopic necrosectomy rate.

The PPI administration route might be an important confounder as theoretically the orally taken PPI might not reach the duodenum/jejunum, where it is regularly resorbed. The pill might enter the WON via LAMS or might be stuck in the stomach due to gastropareses in severe acute pancreatitis. However, we observed a compound-dependent effect for LAMS occlusion (lansoprazole) and endoscopic necrosectomy (esomeprazole) in our multivariable mixed-effects model-based post hoc analysis. As all centers confirmed that these compounds are not available as intravenous solution in their hospitals, we can assume that the administration route might not confound our findings.

GI bleeding through direct friction of the inner parts of the LAMS is a serious complication and could be affected by PPI intake. The risk of delayed bleeding events caused by the sharp edges of the LAMS is considered a clinically relevant problem 18 . It has been suggested that PPIs may reduce rebleeding rates in upper GI bleeding 19 . Nevertheless, our data do not show any significant differences in bleeding events between the cPPI and nPPI groups. Thus, our data suggest that patients who do not take PPIs are not at higher risk for GI bleeding events caused by LAMS.

Finally, our data point toward higher rates of progression of necrotic collections and more superinfection of WON within the cPPI group, which could be a consequence of higher LAMS occlusion rates in this group of patients. It must be acknowledged, however, that our study was not powered for these secondary outcomes.

We included the placement of pre-emptive coaxial pigtail stents through LAMS as a confounder and not as an outcome. However, our data suggest that the placement of coaxial pigtail stents could lead to a significantly lower LAMS occlusion rate and therefore add to the growing body of evidence suggesting that this pre-emptive measure could be beneficial to prevent occlusions.

Nevertheless, our study has several limitations. Importantly, we provide retrospective and thus only associative data. To this end, this study lacks mechanistic data on the potential underlying cause of the effects observed. Hence, we are only able to speculate that a reduction of gastric acid could be the reason for the higher occlusion and endoscopic necrosectomy rates.

Regarding the endoscopic necrosectomy rates, the participating centers may have employed different standards and indications for necrosectomy, depending on local expertise and practice. This might lead to varying endoscopic necrosectomy rates between centers. In a prospective follow-up trial, clear indications should be defined for all participating sites for performing necrosectomy.

Despite including multiple potential confounders, there might be further factors that could potentially influence occlusion and endoscopic necrosectomy rates, such as WON size, density of necrosis, intensive care unit stay, or organ failure. To the best of our knowledge, there is no existing literature directly associating occlusion and necrosectomy rates with these parameters. In a prospective follow-up trial, however, it might be worth controlling for these potential confounders.

In conclusion, our data suggest that patients who received LAMS for drainage of WON upon pancreatitis and who continuously took PPIs experienced higher rates of LAMS occlusion and required a significantly higher number of endoscopic necrosectomies compared with patients who did not take PPIs concomitantly. At the same time, GI bleeding rates were comparable between these two groups. Hence, our data suggest that continuous intake of PPIs could be disadvantageous in patients receiving LAMS for drainage of WON, and discontinuation of PPIs could be beneficial. Nevertheless, prospective and mechanistic trials are needed to confirm our observations and to further investigate the cause of the effects observed.

## Supplementary material

Supplementary material Zusatzmaterial
