# Supplementary material for: Effect of proton pump inhibitors on occlusion of lumen-apposing metal stents and rate of endoscopic necrosectomies: a Europe-wide multicenter cohort study
Source: Endoscopy. 2025 May 8;57(8):829–38. doi: 10.1055/a-2569-7056 (PMC12307040; doi:10.1055/a-2569-7056)

Supplementary material

Effect of proton pump inhibitors on occlusion of lumen-apposing metal stents and rate of endoscopic necrosectomies: a Europe-wide multicenter cohort study

Jacob Hamm[1], Alzbeta Busana[1], Ahmad Amanzada[1], Alexander Arlt[2,3], Thomas Asendorf[4], Samantha Carswell[5], Ulrike Denzer[6], Louis Elsing[7], Fabian Frost[8], Lucia Guilabert[9], Karim Hamesch[10], Marcus Hollenbach[6,11], Peter Hegyi[12,13,14], Alexander Kleger[15,16], Jan Krivinka[17], Lumir Kunovsky[17,18,19], Christian Meinhardt[2], Veit Phillip[20], Sophie Schlosser-Hupf[21], Simon Sirtl[22], Lukas Welsch[23], Julian Cardinal von Widdern[24], Albrecht Neesse[1], Christoph Ammer-Herrmenau[1] and the PROTOCOL working group*

*PROTOCOL working group:

Georg Beyer[22], Alicia Dürr[21], Premysl Falt[17], Christoph Gerst[25], Albrecht Hoffmeister[7], Peter Jeno Hegyi[14], Thomas Kohlmann[26], Richard Knoop[1], Belen Martinez-Moreno[9], Julia Mayerle[22], Patrick Michl[11], Tobias Mollnow[23], Martina Müller[21], Sanjay Pandanaboyana[5,27], Golo Petzold[1], Jonas Rosendahl[24], Lucas Alexander Schulte[16,28], Hans Seifert[2], Emanuel Steiner-Gager[29], Sabrina Sulzer[1], Ondrej Urban[17], Vincent Dansou Zoundjiekpon[17], Volker Ellenrieder[1]

[1] Department of Gastroenterology, Gastrointestinal Oncology and Endocrinology, University Medical Center Goettingen, Germany

[2] Department of Gastroenterology, University Hospital Oldenburg, Oldenburg, Germany

[3] Department of Gastroenterology, Israelitisches Krankenhaus, Hamburg, Germany

[4] Department of Medical Statistics, University Medical Center Goettingen, Goettingen, Germany

[5] HPB and Transplant Unit, Freeman Hospital, Newcastle Upon Tyne, United Kingdom

[6] Department of Gastroenterology, University Hospital Marburg, Marburg, Germany

[7] Department of Oncology, Gastroenterology, Hepatology and Pneumology, University Hospital Leipzig, Leipzig, Germany

[8] Department of Medicine A, University Medicine Greifswald, Greifswald, Germany

[9] Department of Gastroenterology, Hospital General Universitario Dr. Balmis, ISABIAL (Instituto de Investigación Sanitaria y Biomédica de Alicante), Alicante, Spain

[10] Medical Clinic III, Gastroenterology, Metabolic Diseases and Intensive Care, University Hospital RWTH Aachen, Aachen, Germany

[11] Department of Gastroenterology, University Hospital Heidelberg, Heidelberg, Germany

[12] Translational Pancreatology Research Group, Interdisciplinary Center of Excellence for Research Development and Innovation, University of Szeged, Szeged, Hungary

[13] Institute for Translational Medicine, Medical School, University of Pécs, Pécs, Hungary

[14] Institute of Pancreatic Diseases and Centre for Translational Medicine, Semmelweis University, Budapest, Hungary

[15] Institute of Molecular Oncology and Stem Cell Biology, University Hospital Ulm, Ulm, Germany

[16] Division of Interdisciplinary Pancreatology, Department of Internal Medicine I, Ulm University Hospital, Ulm, Germany

[17] 2nd Department of Internal Medicine – Gastroenterology and Geriatrics, University Hospital Olomouc, Faculty of Medicine and Dentistry, Palacky University Olomouc, Olomouc, Czech Republic

[18] Department of Surgery, University Hospital Brno, Faculty of Medicine, Masaryk University, Brno, Czech Republic

[19] Department of Gastroenterology and Digestive Endoscopy, Masaryk Memorial Cancer Institute, Brno, Czech Republic

[20] Department of Clinical Medicine II, Technical University of Munich, TUM School of Medicine and Health, TUM University Hospital, Munich, Germany

[21] Department of Internal Medicine I, Gastroenterology, Hepatology, Endocrinology, Rheumatology and Infectious diseases, University Hospital Regensburg, Regensburg, Bavaria, Germany

[22] Department of Medicine II, University Hospital LMU Munich, Munich, Germany

[23] Department of Gastroenterology, Diabetology and Infectiology, Klinikum Hanau, Hanau, Germany

[24] Department of Internal Medicine I, Martin-Luther-University Halle-Wittenberg, Halle Halle, Germany

[25] Department of Scientific Law, University Medical Center Goettingen, Goettingen, Germany

[26] Institute for Community Medicine, University Hospital Greifswald, Greifswald, Germany

[27] Population Health Sciences Institute, Newcastle University, Newcastle, United Kingdom

[28] Department of Gastroenterology, District Hospitals, Günzburg-Krumbach, Germany

[29] Department of Internal Medicine II, Gastroenterology and Hepatology, Karl Landsteiner University of Health Sciences, University Hospital St. Pölten, St. Pölten, Austria

Supplementary methods

Sample size calculation

An in-house sample set was used to determine the sample size. In total 80 lumen-apposing metal stents (LAMS) implanted at the University Medical Center Goettingen were analyzed retrospectively. 70 patients received proton pump inhibitors continuously (cPPI) and 10 patients did not receive PPIs concomitant with LAMS placement (nPPI). In 16 (22.9%) patients of the cPPI group a LAMS-occlusion was observed, whereas a LAMS occlusion occurred in 1 (10%) patient of the nPPI group. Given two independent groups with a predicted enrollment ratio of 7:1, a probability of type I error of 0.05 and a power of 80% our power calculation resulted in 640 patients (560 patients for the PPI and 80 patients for the non-PPI group). Power calculation was performed using nQuery v9.2.1.0.

Table 1s Diagnostic approaches for lumen-apposing metal stent (LAMS) – occlusion.

**Variable**	**N = 215**[1]
Gastroscopy	209 / 215 (97%)
Endoscopic ultrasound (EUS)	5 / 215 (2.3%)
Computer tomography (CT)	2 / 215 (0.9%)
Abdominal ultrasound	0 / 215 (0%)
Other	
ERCP	2 / 215 (0.9%)

Table 2s Association of proton pump inhibitors (PPI) intake with partial and total lumen-apposing metal stent (LAMS) occlusion rate controlled for potential confounders using a cumulative link mixed model.

Cumulative link mixed model of factors potentially affecting the LAMS partial and total occlusion rate. Wald's test was performed to test significances (* p<0.05, ** p<0.01, *** p<0.001). Following parameters were used as random effects: patient ID and years of LAMS placement. cPPI = continuous PPI intake, nPPI = no PPI intake, CI = Confidence Interval, BMI = Body mass index, OR = Odds Ratio

Variables	No occlusion	Partial occlusion	Total occlusion	OR	95% CI	p-value
**PPI regimen**						
cPPI	405 (71.2%)	55 (9.7%)	109 (19.2%)			
nPPI	148 (78.3%)	16 (8.5%)	25 (13.2%)	0.63	0.4 - 0.99	**0.044***
**Sex**						
Female	169 (71.6%)	22 (9.3%)	45 (19.1%)			
Male	384 (73.6%)	49 (9.4%)	89 (17.0%)	1	0.69 - 1.45	0.996
**BMI**						
<18	16 (88.9%)	0 (0.0%)	2 (11.1%)			
18-25	215 (77.1%)	16 (5.7%)	48 (17.2%)	1.12	0.23 - 5.47	0.891
26-35	288 (71.8%)	44 (11.0%)	69 (17.2%)	1.19	0.24 - 5.79	0.83
>35	34 (56.7%)	11 (18.3%)	15 (25.0%)	2.05	0.39 - 10.71	0.393
**Age**						
18-29	19 (65.5%)	3 (10.3%)	7 (24.1%)			
30-49	146 (76.8%)	16 (8.4%)	28 (14.7%)	0.55	0.22 - 1.36	0.193
50-70	280 (70.7%)	41 (10.4%)	75 (18.9%)	0.78	0.33 - 1.85	0.575
>70	108 (75.5%)	11 (7.7%)	24 (16.8%)	0.56	0.22 - 1.42	0.223
**Type of pancreatitis**						
Acute pancreatitis	434 (69.2%)	69 (11.0%)	124 (19.8%)			
Recurrent acute pancreatitis	39 (88.6%)	1 (2.3%)	4 (9.1%)	0.31	0.11 - 0.92	**0.034***
Chronic pancreatitis	80 (92.0%)	1 (1.1%)	6 (6.9%)	0.19	0.08 - 0.46	**<0.001*****
**Type of LAMS**						
Hot Axios	377 (73.3%)	52 (10.1%)	85 (16.5%)			
Axios	56 (66.7%)	10 (11.9%)	18 (21.4%)	1.12	0.63 - 1.99	0.707
Spaxus	27 (81.8%)	0 (0.0%)	6 (18.2%)	0.88	0.33 - 2.36	0.801
Hot Spaxus	24 (70.6%)	4 (11.8%)	6 (17.6%)	0.89	0.39 - 2.03	0.784
Nagi	5 (83.3%)	1 (16.7%)	0 (0.0%)	0.59	0.06 - 5.47	0.64
Hanaro	44 (84.6%)	1 (1.9%)	7 (13.5%)	0.69	0.3 - 1.59	0.388
Other	16 (59.3%)	2 (7.4%)	9 (33.3%)	2.85	1.16 - 7.01	**0.022***
Not available	4 (50.0%)	1 (12.5%)	3 (37.5%)			
**Pigtails through LAMS**						
Yes	161 (84.3%)	6 (3.1%)	24 (12.6%)	0.5	0.31 - 0.8	**0.004****
No	386 (69.8%)	60 (10.8%)	107 (19.3%)			
**Other gastric pH modulators**						
Yes	17 (70.8%)	4 (16.7%)	3 (12.5%)			
No	536 (73.0%)	67 (9.1%)	131 (17.8%)	0.96	0.35 - 2.65	0.931

Table 3s Association of proton pump inhibitors (PPI) related factors and lumen-apposing metal stent (LAMS) occlusion using a multiple logistic mixed effects model.

Multiple logistic mixed effects model of PPI related factors potentially affecting the LAMS occlusion rate. Wald's test was performed to test significances (* p<0.05, ** p<0.01, *** p<0.001). Following parameters were used as random effects: record id and years of LAMS placement. CI = Confidence Interval, OR = Odds Ratio

Variables	Event Rate	OR	95% CI	p-value
**PPI drug**				
Omeprazole	12 / 65 (18%)			
Esomeprazole	25 / 71 (35%)	1.90	0.74-4.89	0.2
Pantoprazole	144 / 558 (26%)	1.22	0.58-2.56	0.6
Lansoprazole	10 / 13 (77%)	14.1	2.65-74.5	**0.002****
**PPI dose**				
Half/ Standard	114 / 418 (27%)			
Double/ more than double	77 / 289 (27%)	0.92	0.61-1.39	0.7
**Actively stopping PPI**				
Yes	18 / 64 (28%)			
No	168 / 627 (27%)	0.73	0.38-1.39	0.3
Not available	5 / 16 (31%)			
**Change of PPI dose**				
Yes	41 / 140 (29%)			
No	139 / 525 (26%)	0.84	0.53-1.35	0.5
Not available	11 / 42 (26%)			
**PPI prescription**				
Prior to LAMS-application	165 / 613 (27%)			
Upon LAMS-application	23 / 76 (30%)	1.41	0.78-2.54	0.3
Not available	3 / 18 (17%)			
**PPI intake regimen**				
Continuous intake	171 / 574 (30%)			
Intermittent intake	20 / 133 (15%)	0.42	0.23-0.77	**0.005****

Table 4s Association of proton pump inhibitors (PPI) related factors and endoscopic necrosectomy rate (EN) using multiple negative binomial mixed effects model.

Multiple negative binomial mixed effects model for PPI related factors potentially affecting EN rate. Significances (* p<0.05, ** p<0.01, *** p<0.001). Following parameters were used as random effects: record id and years of LAMS placement. CI = Confidence Interval, IRR = Incidence ratio rate

Variables	IRR	95% CI	p-value
**PPI compound**			
Omeprazole			
Esomeprazole	2.06	1.48-2.85	**<0.001*** **
Pantoprazole	1.32	0.99-1.75	0.058
Lansoprazole	1.38	0.77-2.50	0.3
**PPI dose**			
Half/ Standard			
Double/ More than double	1.17	1.01-1.35	**0.039***
**Actively stopping PPI**			
Yes			
No	1.20	0.92-1.55	0.2
Not available	0.80		
**Change of PPI dose**			
Yes			
No	0.85	0.72-1.00	**0.046***
Not available	0.67		
**PPI prescription**			
Prior to LAMS-application			
Upon LAMS-application	1.02	0.82-1.28	0.8
Not available	0.77		
**PPI intake regimen**			
Continuous intake			
Intermittent intake	0.88	0.71-1.09	0.2
Not available	1.01		

[1]CI = Confidence Interval

Table 5s Association of different proton pump inhibitors (PPI) compound and endoscopic necrosectomy rate (EN) using pairwise comparison of a multiple negative binomial mixed effects model.

Pairwise comparison of different PPI compounds from multiple negative binomial mixed effects model. Results are averaged over the levels of: PPI dose, actively stopped PPI, change in PPI doses, PPI prescription, PPI intake regimen. Following parameters were used as random effects: record id and years of LAMS placement. P value adjustment was conducted with Tukey method for comparing a family of four estimates (* p<0.05, ** p<0.01, *** p<0.001). CI = Confidence Interval, IRR – Incidence ratio rate.

Groups	OR	95% CI	p-value adj.
Omeprazole - Esomeprazole	2.06	0.32 - 0.75	**<0.001** ***
Omeprazole - Pantoprazole	1.32	0.52 - 1.1	0.23
Omeprazole - Lansoprazole	1.38	0.33 - 1.57	0.703
Pantoprazole - Esomeprazole	1.56	1.21 - 2.02	**<0.001** ***
Pantoprazole - Lansoprazole	1.05	0.48 - 1.88	0.998
Esomeprazole - Lansoprazole	0.67	0.72 - 3.05	0.489

Table 6s Association of proton pump inhibitors (PPI) and lumen-apposing metal stent (LAMS)-related complications.

Logistic regression with Firth's correction was performed. CI – Confidence interval, OR – Odds ratio, WON = walled off necrosis, cPPI – continuous PPI and nPPI – no PPI intake.

Variables	cPPI	nPPI	OR cPPI/ nPPI (95% CI)
**Gastrointestinal bleeding**	43/582 (7.4%)	16/195 (8.2%)	1.14 (0.61 – 2.02)
**WON Progress**	12/582 (2.1%)	1/195 (0.5%)	0.35 (0.04-1.47)
**Superinfection of WON**	21/582 (4.3%)	6/195 (3.1%)	0.75 (0.29-1.7)
**Sepsis**	17/582 (2.9%)	3/195 (3.6%)	1.29 (0.51-2.97)

Table 7s Association of proton pump inhibitors (PPI) intake regimen and duration of indwelling lumen-apposing metal stent (LAMS) using a multiple negative binomial mixed effects model.

Multiple negative binomial mixed-effects model of factors potentially affecting the duration of indwelling LAMS days. Following parameters were used as random effects: record id and years of LAMS placement. CI = Confidence Interval, BMI = Body mass index, IRR = Incidence ratio rate

**Variables**	**IRR**	**95% CI**[1]	**p-value**
**PPI intake regimen**			
Continuous intake	—	—	
No intake	0.91	0.80-1.05	0.2
**Sex**			
Female	—	—	
Male	1.00	0.88-1.14	>0.9
**Type of pancreatitis**			
Acute pancreatitis	—	—	
Recurrent acute pancreatitis	0.85	0.65-1.11	0.2
Chronic pancreatitis	0.90	0.74-1.09	0.3
**Type of LAMS**			
Hot Axios	—	—	
Axios	1.19	0.97-1.45	0.089
Spaxus	1.13	0.85-1.50	0.4
Hot Spaxus	3.99	3.29-4.84	**<0.001*** **
Nagi	1.72	0.89-3.31	0.10
Hanaro	1.06	0.85-1.33	0.6
Other	0.90	0.60-1.35	0.6
Not available	0.74		
**Pigtails through LAMS**			
No	—	—	
Yes	1.56	1.37-1.78	**<0.001*** **
**Access way**			
Transgastric access	—	—	
Transduodenal access	0.94	0.76-1.17	0.6
Other access	2.05	1.13-3.72	**0.019***
Not available	1.71		
**LAMS diameter**			
15mm - 20mm	—	—	
> 10mm < 15mm	1.20	1.03-1.41	**0.021***
Not available	1.05		
**Age**			
18-29	—	—	
30-49	0.73	0.52-1.01	0.058
50-70	0.78	0.56-1.07	0.12
>70	0.71	0.51-1.00	0.052
**BMI**			
<18	—	—	
18-25	0.83	0.59-1.17	0.3
26-35	0.93	0.66-1.33	0.7
>35	0.87	0.58-1.30	0.5
**Other gastric pH modulators**			
Yes	—	—	
No	0.72	0.54-0.96	**0.023***

Thieme

Fig. 1s Cumulative incidences of lumen-apposing metal stents (LAMS) occlusions for continuous proton pump inhibitors (cPPI) and non-PPI group (nPPI).

Censored cases were marked with ("+"). P-value was calculated by Gray's test.

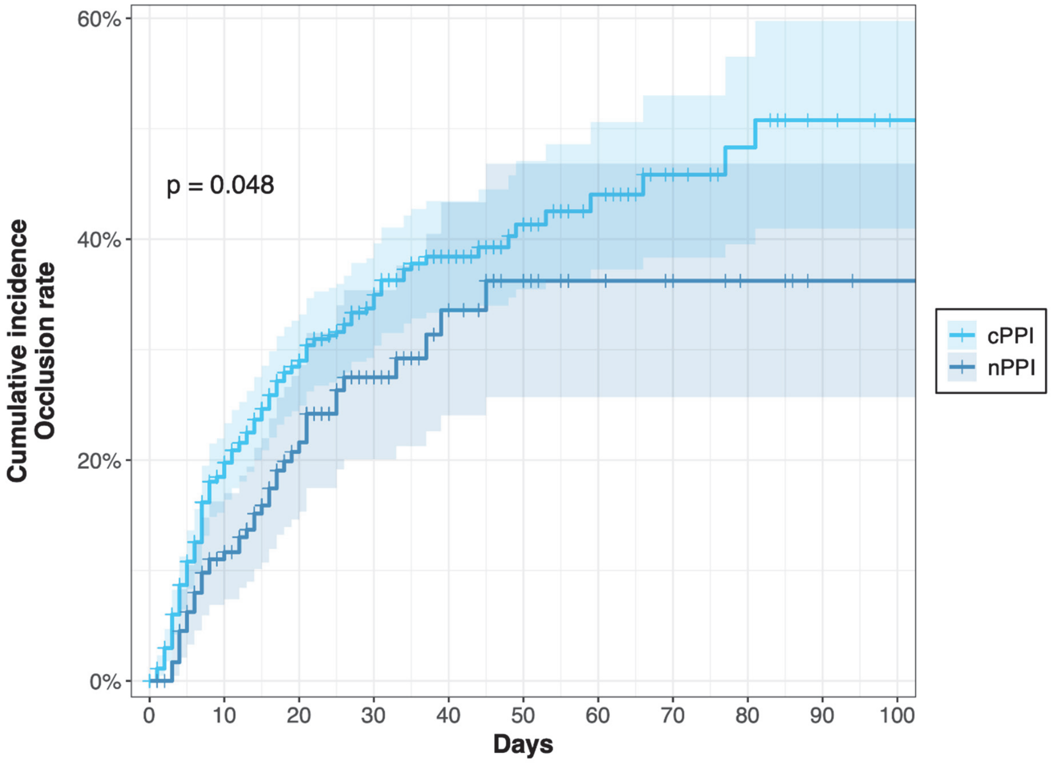

	0	10	20	30	40	50	60	70	80	90	100
**Continuous intake**											
At Risk	577	415	301	198	119	93	74	64	58	53	50
Events	0	101	139	157	164	167	169	170	171	172	172
**No intake**											
At Risk	191	150	104	63	42	30	22	20	17	14	13
Events	0	20	33	39	42	43	43	43	43	43	43

Fig. 2s Histogram days to lumen-apposing metal stent removal.

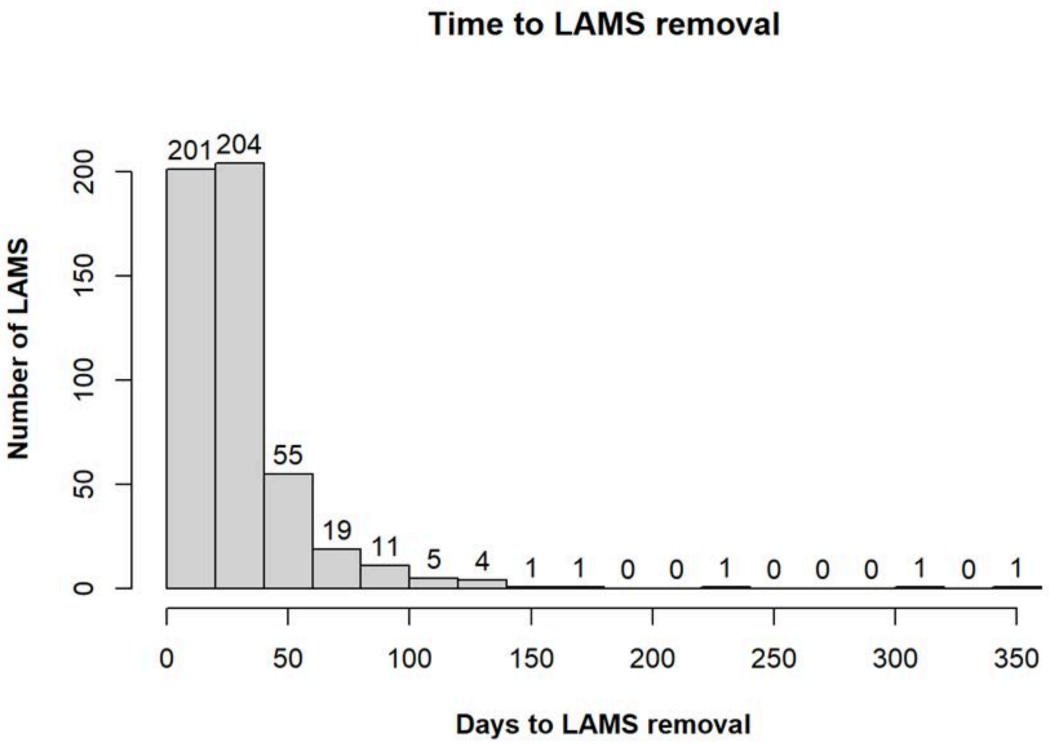

Supplement: Supplementary file 1 — Supplementary material [file 24336supmat_10-1055-a-2569-7056.pdf]
